# Null phenotype non-Hodgkin Lymphoma presenting as a large chest wall mass: case report

**Published:** 2012-12-12

**Authors:** Sakina Sekkate, Mouna Kairouani, Nawfal Mellas, Hanane El Kabbaj, Hind M'rabti, Hassan Errihani

**Affiliations:** 1 Department of Clinical Oncology, Hassan II Hospital, Fes, Morocco; 2 Department of Radiotherapy, National Institute of Oncology, 10100, Rabat, Morocco

**Keywords:** Lymphoma, null phenotype, chest tumor, diagnosis

## Abstract

Tumors of chest wall represent a variant entity. Most of them arise from metastasis of malignant tumors or from local invasion by contiguity. However, non-Hodgkin's lymphomas of the chest wall are extremely rare; only a few cases have been reported in the literature. We report a case about a Moroccan woman, with non-Hodgkin null phenotype lymphoma of the chest, treated successfully with CHOP (cyclophosphamide, Doxorubicin, Vincristin and prednisone) followed by local radiotherapy.

## Introduction

Chest wall tumors arise from a large variety of malignant and benign etiologies, and present a diagnostic challenge for the clinician. The classification of chest wall tumors includes primary tumors, adjacent tumors with local invasion, metastatic lesions, and non neoplastic disease [1, 2]. Non-Hodgkin lymphoma presenting as a solitary chest wall mass is not frequently seen [1].

## Patient and observation

**Figure 1:**
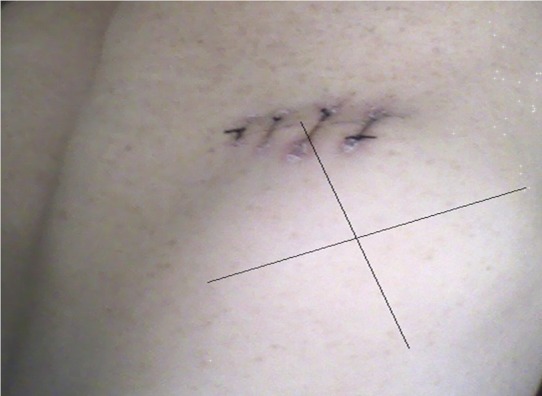
The chest wall tumor measuring 10 cm in diameter

**Figure 2:**
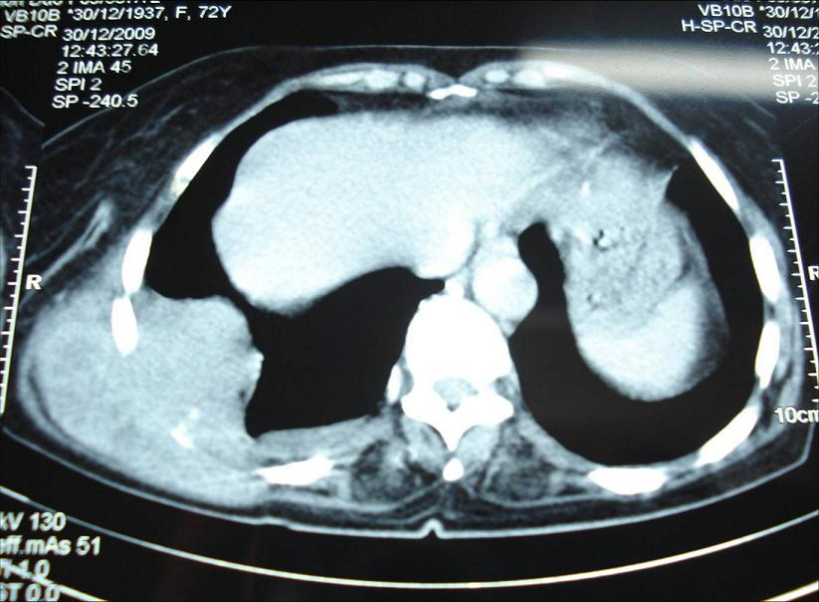
Computed tomography scan of the chest:Soft tissue mass lesion in posterior chest wall with thorax extension accompanied by pleural thickening

A 60-year-old woman with no history of tuberculosis, pyothorax or artificial pneumothorax therapy, presented 4 months before her admission an asthenia, fever, chills, and night sweats. Physical examination revealed a mass of 10 cm localised in lateral chest wall (Figure 1). ignificant laboratory findings were as follows: haemoglobin: 9,3g/dL, erythrocyte sedimentation rate: 60 mm/h, LDH rate: 620 UI/ml. Computed tomographic scan confirmed the localisation in chest wall with invasion of the ribs (Figure 2).

A biopsy of the chest tumor was performed and revealed a non-Hodgkin lymphoma with null phenotype. Immunohistochemical staining of the specimen was positive for leukocyte common antigen, CD20, and Ki67 and negative for CD3, vimentin, desmin, chromogranin, neuron specific enolase, CD99, and actin.

The patient received four cycles of chemotherapy CHOP 21 (Cyclophosphamide 750 mg/m2 Intraveinously (IV) at day1, doxorubicin 50 mg/m2 IV at day1, vincristine 1, 4 mg/m2 with a maximum total dose of 2 mg IV at day1 and prednisone 40 mg/m2/day per os from day 1 to 5) followed by local radiotherapy, total dose was 40 Gy delivered as 2Gy daily fractions, 5 day per week. She had a complete response maintained for more than 7 months.

## Discussion

The majority of chest wall lesions arise from metastasis or invasion from adjacent malignancies [1]. Among primary chest wall tumors, chest wall lymphoma is extremely rare, accounting for less than 2% of chest wall soft tissue tumors [2, 3]. Few cases of primary malignant lymphoma, arising from the sternum, the pleura or the rib have been reported. Primary bone lymphoma can result from any part of the skeleton. The most common tumor sites are the long bones (50% extremities), followed by the axial skeleton (44%) [2].

Our patient had a primary non-Hodgkin lymphoma in the ribs. Primary lymphoma of the bone (PLB), which was first reported by Oberling, [4] accounts for approximately 4.7% of extranodal lymphomas. The majority of cases occur in long bones, spine, or pelvic, and rib involvement is rare (2% to 15% of cases of PLB). According to a review by Ostrowski and associates, the mean age at presentation was 46 years with male predominance. The most frequent symptoms are pain, swelling, fever, night sweats, and body weight loss, as in other lymphoma conditions. [5] Because imaging study of chest wall tumor suggesting PLB is not diagnostic, a histologic specimen is needed [6].

The treatment in general is conservative, based on the stage of disease. PBL therapy has envolved from radiotherapy as a standalone strategy (since the 1960s) to combined modality therapy (chemotherapy and radiotherapy) [4]. There have also been a few reports of patients treated with chemotherapy alone [2, 3]. There is no definitive therapeutic strategy. Local radiation is recommended, and additional systemic chemotherapy seems to be effective [6]. Recently, Mavrogenis and al [7], reported the role of surgery for heamatologic neoplasms of bone about 205 patients treated from 1985 to 2009, the survival to death was improved after wild resection for lymphoma and plasmacytoma. But no statistical difference was noted in survival to local recurrence between wide and intralesional surgery.

## Conclusion

Primary chest lymphoma is rare; therefore, the therapeutic management is unclear. Data on the treatment and evolution of this disease should be reported.
